# Phenylephrine and the risk of atrial fibrillation in critically ill patients: a multi-centre study from eICU database

**DOI:** 10.3389/fphar.2025.1478961

**Published:** 2025-03-26

**Authors:** ZhiMing Huang, Weichao Li, WeiXian Xie, Gu xun-hu, Heng Li

**Affiliations:** ^1^ Department of Anesthesiology, Affiliated Qingyuan Hospital (Qingyuan People’s Hospital), Guangzhou Medical University, Qingyuan, Guangdong, China; ^2^ Department of Neurology, The Second Affiliated Hospital of Nanchang University, Nanchang, Jiangxi, China

**Keywords:** Vasopressors, New atrial fibrillation, risk, phenylephrine, critically ill patients

## Abstract

**Background:**

Vasopressors are vital for maintaining blood pressure in critically ill patients, though they carry risks like irregular heartbeats and impaired cardiac oxygen balance. Existing studies have not definitively proven that phenylephrine triggers new atrial fibrillation (AF).

**Aims:**

This study was designed to assess pharmacological associations between phenylephrine utilization and new AF occurrence risk.

**Methods:**

This multicenter retrospective study analyzed eICU database records. Propensity score matching (PSM) balanced baseline confounders. Cox regression models (unadjusted/adjusted) assessed phenylephrine-AF associations.

**Results:**

In this cohort encompassing 51,294 critically ill adults (mean age 62.4 ± 16.6 years; 53.5% male), propensity score matching established comparable cohorts: 2,110 phenylephrine-exposed patients and 6,330 matched controls. The analysis revealed a clinically significant disparity in new AF incidence, with phenylephrine-exposed patients demonstrating a 10.5% event rate (282/2,673) *versus* 4.9% (2,395/48,621) in non-exposed counterparts (p < 0.001). Multivariable-adjusted Cox proportional hazards models identified a 29% elevated risk of new AF associated with phenylephrine administration (aHR, 1.29; 95%CI, 1.05–1.58). Notably, this association remained robust across multiple sensitivity analyses employing alternative matching methodologies and covariate adjustments.

**Conclusion:**

This evidence positions phenylephrine as a modifiable new AF risk factor in critical care, supporting risk-aware vasopressor selection through benefit-harm analysis.

## Introduction

Post-procedure AF is a common occurrence in adults with severe arrhythmogenesis (Karamchandani et al., 2020), showing more links to increased mortality and stroke risks (AlTurki et al., 2020). While catecholamine vasopressors are frequently employed for low blood pressure during surgery; however, all such devices come with side effects, like cardiac rhythm issues and myocardial ischemia (Schmittinger et al., 2012). Catecholamines modulate cardiac electromechanics via β-adrenoceptor-driven Ca^2+^ regulation. Elevated concentrations exhibit arrhythmogenic properties, mechanistically contributing to atrial fibrillation pathogenesis (Chelazzi et al., 2011; Workman, 2010). Under intra- and postoperative conditions, sympathetic excess, inflammatory cytokines (Ramlawi et al., 2007), metabolic dysregulation, impaired metabolic balance and compromised myocardial energy (Wu et al., 2015) have been proposed to possibly contribute to the development of AF (Maesen et al., 2012; Zakkar et al., 2015). Previous randomized controlled trials have shown a lower occurrence of AF in patients receiving vasopressin (63.8% vs. 82.1%) than in patients receiving norepinephrine for the treatment of vasoplegic shock after cardiac surgery (Hajjar et al., 2017). Additionally, phenylephrine, by stimulating α1-adrenergic receptors, exerts significant effects on the autonomic nervous system. It increases systemic vascular resistance and afterload, which can lead to a reduction in cardiac output. These hemodynamic changes may contribute to atrial stretch, elevated left atrial pressure, and sympathetic overactivation, all of which are well-established mechanisms that increase the risk of AF. Current evidence fails to establish causality between catecholamine vasopressors and postoperative AF. This investigation specifically examined their potential to induce new AF in perioperative settings.

## Methods

### Observational cohort

This retrospective multicenter analysis utilized de-identified clinical data from the eICU Collaborative Research Database (v2.0), comprising 139,367 critically ill patients with 200,859 ICU admissions across 208 American hospitals (2014-2015) (O'Halloran et al., 2020; Pollard et al., 2018). One investigator (WCL) secured authorized access to the clinical data repositories; completion of the CITI Program certification (Record ID: 13586991) validated compliance with human subjects research ethics standards. Ethical approval was waived under HIPAA Safe Harbor provisions (certification #1031219-2) due to pre-existing de-identification. The study adhered to STROBE-RECORD reporting guidelines for observational research using routinely collected health data (von Elm et al., 2007; Benchimol et al., 2015).

### Primary outcome and intervention

The primary outcome of the research focused on newly detected atrial fibrillation (AF) cases emerging within 30 days of ICU admission. This clinical condition was characterized by ECG-confirmed atrial fibrillation possibly accompanied by manifestations such as angina symptoms, acute cardiac insufficiency, sustained hypotension, or necessitating therapeutic interventions including heart rate regulation medication, rhythm control agents, or electrical cardioversion. Diagnostic verification was achieved through International Classification of Diseases coding (ICD, I48). Regarding pharmacological interventions, catecholamine vasopressor administration - encompassing agents like norepinephrine, epinephrine, phenylephrine, dopamine, or milrinone - was recorded either within the initial 24-h ICU period or during clinical procedures.

### Sample and sources

The analytic cohort was derived through systematic screening of all hospitalized individuals within the database. Exclusion parameters comprised: 1) intensive care unit (ICU) length of stay <48 h or >30 days, 2) incomplete pharmacological documentation, 3) pediatric populations (age <18 years), and 4) documented pre-existing atrial fibrillation.

### Data collection and definitions

Programmatic data extraction via SQL protocols retrieved multi-domain clinical parameters, including: 1) Demographic profiles with comorbidity classifications per ICD-9-CM diagnostic coding standards; 2) Pharmacotherapeutic regimens; 3) Essential laboratory parameters captured within the initial 24-h ICU monitoring window. Critical illness severity was quantified through validated scoring instruments: Simplified Acute Physiology Score II (SAPS-II) and Acute Physiology and Chronic Health Evaluation IV (APACHE-IV). Prognostic information including mortality in ICUs and hospitals is also extracted. Non-cardiac procedures were defined as surgical or medical interventions that do not directly involve the heart or major cardiovascular structures. These include, but are not limited to, orthopedic surgeries (e.g., joint replacements), abdominal surgeries (e.g., colectomy, cholecystectomy), thoracic surgeries (e.g., lung resections), neurosurgical procedures, and major urological or gynecological operations.

### Propensity score matching (PSM) and sensitivity testing

To address selection bias, propensity score matching (PSM) was executed via multivariable logistic regression incorporating covariates from Table 1; Supplementary Figure 1, estimating phenylephrine exposure likelihood. A 1:3 nearest-neighbor algorithm established balanced cohorts (intervened vs. non-intervened). Analytical rigor was ensured through: 1) Sensitivity analyses with alternative PSM specifications (Parsons and Ovation Research Group, 2001); 2) Multivariable-adjusted logistic regression identifying independent predictors; 3) Temporal stratification via Cox proportional hazards modeling; 4) Pharmacodynamic evaluation of phenylephrine administration timing (perioperative/ICU phases).

**TABLE 1:** Basic features of Phenylephrine and Nonphenylephrine groups from Pre-/Post-Matching.

	Propensity score weighting						
		Before			After		
	Overall	Non phenylephrine	Phenylephrine	SMD	Non phenylephrine	Phenylephrine	SMD
	N = 51294	N = 48621	N = 2673		N = 6330	N = 2110	
Basic Information							
Age (mean (SD))	62.43 (16.62)	62.32 (16.71)	64.52 (14.73)	0.14	64.71 (15.45)	64.42 (14.93)	0.019
Male (%)	27427 (53.5)	25877 (53.2)	1550 (58.0)	0.097	3621 (57.2)	1204 (57.1)	0.018
BMI (mean (SD))	84.00 (27.23)	83.97 (27.34)	84.39 (25.11)	0.016	84.00 (27.95)	83.68 (24.84)	0.012
Race (%)				0.09			0.035
African American	6902 (13.5)	6600 (13.6)	302 (11.3)		731 (11.5)	244 (11.6)	
Caucasian	38992 (76.0)	36878 (75.8)	2114 (79.1)		5040 (79.6)	1663 (78.8)	
Hispanic	1886 (3.7)	1794 (3.7)	92 (3.4)		195 (3.1)	72 (3.4)	
Asian	663 (1.3)	628 (1.3)	35 (1.3)		67 (1.1)	28 (1.3)	
Native American	407 (0.8)	395 (0.8)	12 (0.4)		21 (0.3)	6 (0.3)	
Other/Unknown	2444 (4.8)	2326 (4.8)	118 (4.4)		276 (4.4)	97 (4.6)	
Heart rate (mean (SD))	89.11 (20.94)	88.96 (20.82)	91.75 (22.76)	0.128	90.81 (21.62)	91.07 (22.63)	0.012
Systolic blood (mean (SD))	124.18 (29.07)	124.96 (28.98)	109.97 (26.87)	0.536	112.09 (27.92)	112.11 (26.71)	0.001
Diastolic blood (mean (SD))	68.70 (18.45)	69.05 (18.36)	62.31 (18.85)	0.362	63.34 (17.97)	63.28 (18.72)	0.003
APACHE score (mean (SD))	57.33 (25.47)	56.38 (24.74)	74.58 (31.69)	0.64	69.41 (28.54)	69.51 (28.20)	0.003
APS score (mean (SD))	45.82 (23.76)	44.91 (22.96)	62.37 (30.93)	0.641	57.10 (27.44)	57.38 (27.26)	0.01
Respiratory rate (mean (SD))	19.31 (7.12)	19.32 (7.11)	19.17 (7.36)	0.021	19.01 (7.49)	19.11 (7.32)	0.013
Spo_2_ (mean (SD))	97.00 (4.14)	97.01 (4.06)	96.77 (5.48)	0.051	97.08 (3.96)	96.94 (5.03)	0.029
Temperature (mean (SD))	36.71 (0.80)	36.72 (0.79)	36.51 (1.03)	0.23	36.61 (0.99)	36.61 (0.96)	0.003
Stay, day (mean (SD))	7.94 (5.30)	7.84 (5.25)	9.68 (5.90)	0.329	9.41 (5.89)	9.58 (5.75)	0.028
ICU duration, day (mean (SD))	3.54 (3.25)	3.45 (3.13)	5.32 (4.66)	0.473	4.82 (4.45)	5.02 (4.29)	0.045
Thoracic surgery (%)	3782 (7.4)	3437 (7.1)	345 (12.9)	0.196	722 (11.4)	252 (11.9)	0.017
Cardiac surgery (%)	2165 (4.2)	1686 (3.5)	479 (17.9)	0.481	843 (13.3)	253 (12.0)	0.04
ICU type (%)				0.411			0.094
Med-Surg ICU	25501 (49.7)	24454 (50.3)	1047 (39.2)		2583 (40.8)	899 (42.6)	
Neuro ICU	5384 (10.5)	5178 (10.6)	206 (7.7)		592 (9.4)	185 (8.8)	
CCU-CTICU	5169 (10.1)	4762 (9.8)	407 (15.2)		867 (13.7)	288 (13.6)	
MICU	4871 (9.5)	4715 (9.7)	156 (5.8)		364 (5.8)	134 (6.4)	
SICU	3925 (7.7)	3508 (7.2)	417 (15.6)		880 (13.9)	244 (11.6)	
Cardiac ICU	3083 (6.0)	2924 (6.0)	159 (5.9)		429 (6.8)	135 (6.4)	
CTICU	1776 (3.5)	1628 (3.3)	148 (5.5)		378 (6.0)	124 (5.9)	
CSICU	1585 (3.1)	1452 (3.0)	133 (5.0)		237 (3.7)	101 (4.8)	
Ventilation (%)	3777 (7.4)	3316 (6.8)	461 (17.2)	0.325	946 (14.9)	321 (15.2)	0.008
Comorbidities							
Asthma (%)	3683 (7.2)	3528 (7.3)	155 (5.8)	0.059	373 (5.9)	133 (6.3)	0.017
CKD (%)	5998 (11.7)	5666 (11.7)	332 (12.4)	0.024	728 (11.5)	266 (12.6)	0.034
Dialysis (%)	1908 (3.7)	1801 (3.7)	107 (4.0)	0.016	210 (3.3)	85 (4.0)	0.038
COPD (%)	7697 (15.0)	7325 (15.1)	372 (13.9)	0.033	916 (14.5)	304 (14.4)	0.002
Diabetes (%)	7632 (14.9)	7322 (15.1)	310 (11.6)	0.102	717 (11.3)	265 (12.6)	0.038
HF (%)	6745 (13.1)	6369 (13.1)	376 (14.1)	0.028	804 (12.7)	280 (13.3)	0.017
Hypertension (%)	25803 (50.3)	24300 (50.0)	1503 (56.2)	0.125	3522 (55.6)	1166 (55.3)	0.008
Hyperthyroidism (%)	178 (0.3)	168 (0.3)	10 (0.4)	0.005	30 (0.5)	8 (0.4)	0.015
MI (%)	4711 (9.2)	4389 (9.0)	322 (12.0)	0.098	741 (11.7)	243 (11.5)	0.006
Peripheral vascular disease (%)	2348 (4.6)	2196 (4.5)	152 (5.7)	0.053	352 (5.6)	117 (5.5)	0.001
Respiratory failure (%)	909 (1.8)	872 (1.8)	37 (1.4)	0.033	96 (1.5)	32 (1.5)	<0.001
Stroke (%)	4041 (7.9)	3822 (7.9)	219 (8.2)	0.012	539 (8.5)	178 (8.4)	0.003
Sepsis (%)	8939 (17.4)	8274 (17.0)	665 (24.9)	0.194	1548 (24.5)	499 (23.6)	0.019
Medication							
Beta-blockers (%)	10459 (20.4)	9848 (20.3)	611 (22.9)	0.063	1328 (21.0)	454 (21.5)	0.013
Dexmedetomidine (%)	1521 (3.0)	1341 (2.8)	180 (6.7)	0.188	316 (5.0)	82 (3.9)	0.054
ARB/ACEI (%)	7852 (15.3)	7559 (15.5)	293 (11.0)	0.136	659 (10.4)	236 (11.2)	0.025
Aspirin (%)	14726 (28.7)	13817 (28.4)	909 (34.0)	0.121	2045 (32.3)	701 (33.2)	0.02
CCB (%)	4873 (9.5)	4689 (9.6)	184 (6.9)	0.1	442 (7.0)	163 (7.7)	0.028
Clopidogrel (%)	4808 (9.4)	4606 (9.5)	202 (7.6)	0.069	464 (7.3)	174 (8.2)	0.034
Diuretic (%)	13654 (26.6)	12780 (26.3)	874 (32.7)	0.141	1946 (30.7)	644 (30.5)	0.005
Statins (%)	9407 (18.3)	8935 (18.4)	472 (17.7)	0.019	1074 (17.0)	371 (17.6)	0.016
MgSo_4_ (%)	15334 (29.9)	14260 (29.3)	1074 (40.2)	0.229	2456 (38.8)	845 (40.0)	0.026
Opioid (%)	25199 (49.1)	23708 (48.8)	1491 (55.8)	0.141	3362 (53.1)	1139 (54.0)	0.017
Fluids infusion							
Intake total (mean (SD))	4693.54 (10341.36)	4427.65 (9962.96)	9530.08 (14903.04)	0.403	6425.93 (10790.71)	7057.71 (11182.58)	0.057
Output total (mean (SD))	3267.88 (4439.94)	3182.52 (4255.35)	4820.60 (6810.40)	0.288	3958.45 (5818.78)	3953.59 (5548.74)	0.001
Vasoactive drugs							
Vasopressin (%)	1389 (2.7)	926 (1.9)	463 (17.3)	0.542	395 (6.2)	184 (8.7)	0.094
Epinephrine (%)	859 (1.7)	532 (1.1)	327 (12.2)	0.458	149 (2.4)	103 (4.9)	0.136
Norepinephrine (%)	6776 (13.2)	5791 (11.9)	985 (36.8)	0.607	1739 (27.5)	618 (29.3)	0.04
Dopamine (%)	1414 (2.8)	1097 (2.3)	317 (11.9)	0.382	414 (6.5)	147 (7.0)	0.017
Milrinone (%)	428 (0.8)	283 (0.6)	145 (5.4)	0.287	85 (1.3)	45 (2.1)	0.06

Abbreviations: AF, atrial fibrillation; CCB, calcium channel blocker; ARB, angiotensin receptor blocker; ACEI, angiotensin converting enzyme inhibitor; Med-Surg ICU, Medical-Surgical ICU; SICU, Surgical ICU; CSICU, Cardiovascular Surgery ICU; CCU-CTICU, Cardiology care unit-Cardiothoracic ICU; CTICU, Cardiothoracic ICU; MICU, Medical ICU; SAPS II, simplified acute physiology; APACHE IV, Acute Physiology and Chronic Health Evaluation IV.

### Statistical analyses

Sample size determination incorporated established atrial fibrillation prevalence estimates (4.6% baseline incidence), projecting detection of 46 AF cases among 1,000 enrollees. The power calculation framework mandated 7,107 participants to achieve 0.8 C-statistic reliability with 47 predictor variables, maintaining 0.05 precision in adjusted R^2^ estimation (Riley et al., 2020).

Continuous parameters were stratified by distribution normality (Kolmogorov-Smirnov test) and analyzed via parametric or nonparametric tests, presented as mean ± SD. Categorical variables were expressed as frequency distributions (%) with χ^2^ analysis. Cox proportional hazards models (unadjusted/adjusted) quantified phenylephrine-AF risk associations through hazard ratios (95% CIs). Statistical significance threshold was set at α = 0.05 (two-tailed), with analyses executed in R 4.2.1 (R Foundation) and SPSS v25.

## Results

### Patient characteristics

**FIGURE 1:**
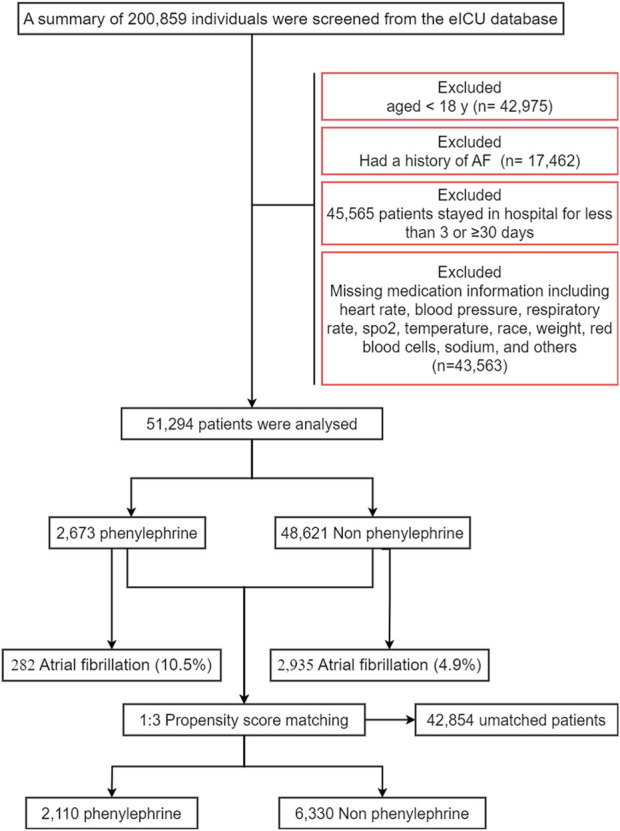
Flowchart of subject selection.

A total of 51,294 participants were eligible for the trial (Figure 1). The median (SD) age was 62.43 (16.62) years, and 53.5% were male. The higher proportion of older, males, higher APACHE or APS score, cardiac or thoracic surgery, ventilation, co-morbidities, drug treatments, and vasoactive drugs (Table 1).

The propensity-matched analysis included 8,440 critically ill patients (mean age 64.6 ± 15.3 years; 42.8% female), comprising 2,110 phenylephrine-intervened cases and 6,330 matched controls. Demographic parity was demonstrated through standardized mean differences <0.10 across all baseline parameters (Table 1), with detailed covariate distributions visualized in Supplementary Figure S1. This rigorous matching protocol ensured comparable group characteristics for subsequent outcome analyses.

### Risk of developing new-onset AF

The phenylephrine cohort demonstrated significantly higher atrial fibrillation incidence (282 cases, 10.5%) compared to non-intervened counterparts (2,395 cases, 4.9%). Multivariable-adjusted Cox regression analysis identified a 29% increased AF risk with phenylephrine intervention (aHR, 1.29; 95% CI 1.05–1.58; p = 0.017), as detailed in Table 2. Supplementary analyses revealed differential AF risk profiles among antihypertensive agents (Supplementary Table S1), with comparative pharmacovigilance analysis identifying phenylephrine as carrying elevated postoperative AF risk relative to norepinephrine (Supplementary Table S2).

**TABLE 2:** Association of phenylephrine exposure with new atrial fibrillation: Unadjusted and adjusted models via cox/logistic regression including temporal stratification and propensity-score matching.

Analysis		
No. Of events/no. Of patients at risk (%)	New-onset AF	P value
Phenylephrine group	282/2673(10.5%)	
No Phenylephrine group	2395/48621(4.9%)	
Before propensity score matching		
Crude analysis-hazard ratio (95% Cl)	1.52(1.34–1.72)	<0.001
Multivariable analysis-adjusted hazard ratio (95% Cl)		
All phenylephrine^**a1**^	1.29(1.05–1.58)	0.017
Intraoperative phenylephrine^**a2**^	1.62(1.39–1.90)	<0.001
ICU phenylephrine^**a3**^	1.45(1.24–1.69)	<0.001
Multivariable time-varying analysis-adjusted hazard ratio (95% Cl)^**b**^	1.56(1.37–1.81)	<0.001
Logistic regression analysis-adjusted odds ratio (95% Cl)^**c**^	1.79(1.54–2.07)	<0.001
After propensity score matching		
Propensity-score analyses-adjusted hazard ratio (95% Cl)		
With matching^**d1**^	1.51(1.27–1.78)	<0.001
Adjusted for propensity score^**d2**^	1.52(1.34–1.71)	<0.001

a1 The multivariable Cox analysis model incorporating demographic characteristics, comorbidities, current medications, and laboratory tests (Full cohort patients).

a2 The multivariable Cox analysis model incorporating the same covariates.

Patients with phenylephrine use in the ICU were excluded from the analysis.

a3 The multivariable Cox analysis model incorporating the same covariates.

Patients with intraoperative use of phenylephrine were excluded from the analysis.

^b^ The multivariable Cox analysis model included follow-up data as time-varying covariable, and incorporated for the same covariates. All patients were included in the analysis.

^c^ A multivariable Logistic regression model with the same covariates (Full cohort patients).

d1 the univariate Cox proportional hazards model from matching propensity-score. A total of 8440 subjects were included.

d2 The multivariable Cox analysis model incorporating the same covariates and additional the propensity score. A total of 8440 subjects were included.

### Sensitivity analyses

Sensitivity analyses demonstrated differential AF risk profiles based on phenylephrine administration timing: intraoperative exposure exhibited a 62% elevated risk (aHR, 1.62; 95%CI, 1.39–1.90; p < 0.001), while ICU administration showed 45% increased likelihood (aHR, 1.45; 95%CI, 1.24–1.69; p < 0.001). Methodological consistency was confirmed through multivariable approaches - logistic regression (aOR, 1.79; 95%CI, 1.54–2.07; p < 0.001) and time-dependent Cox modeling (aHR, 1.56; 95%CI, 1.37–1.81; p < 0.001). Propensity score refinement strategies, including matched cohort analysis (aHR, 1.51; 95%CI, 1.27–1.78) and inverse probability weighting (aHR, 1.52; 95%CI, 1.34–1.71), revealed congruent risk elevations (all p < 0.001).

**FIGURE 2:**
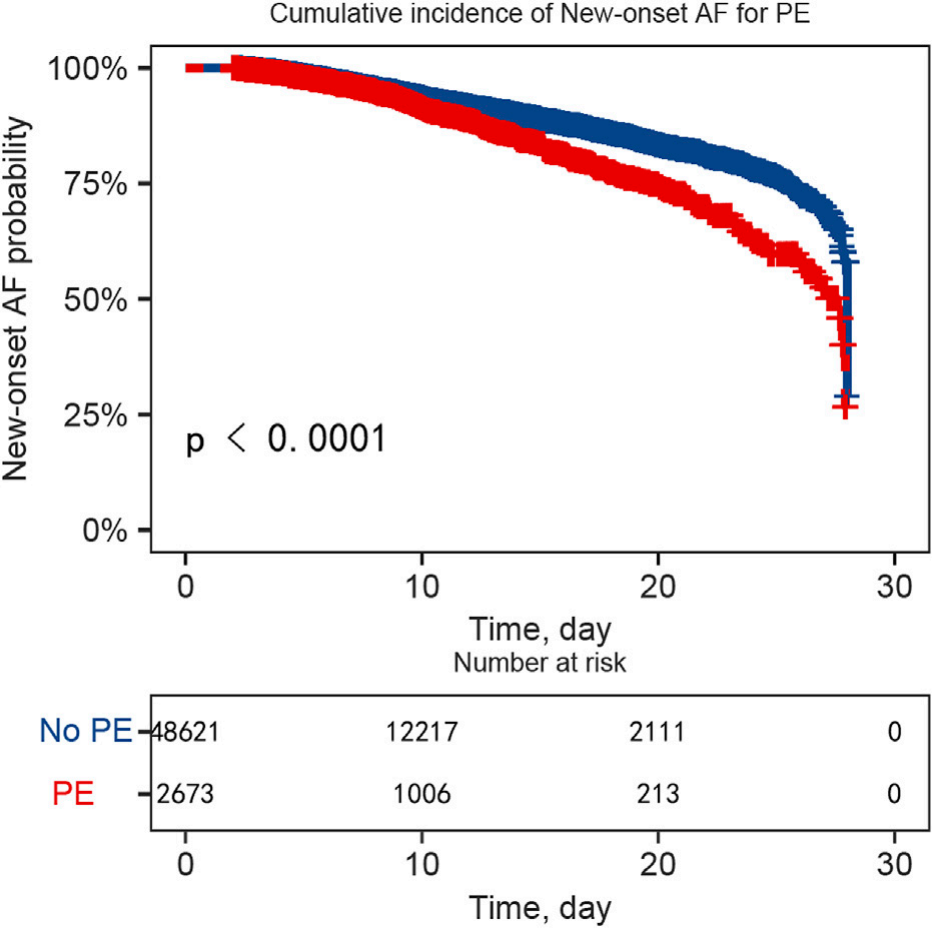
Temporal risk progression of phenylephrine-associated atrial fibrillation.

Temporal risk progression patterns were visualized through cumulative incidence curves: Figure 2 delineates overall phenylephrine-AF associations, while Supplementary Figure S2 provides phase-specific stratification (intraoperative/ICU administration epochs).

## Discussion

This observational study revealed a clinically significant elevation in AF risk among phenylephrine-exposed patients compared to non-exposed counterparts. The pharmacological correlation persisted across multiple analytical frameworks, demonstrating particular robustness in temporal-stratified models assessing intraoperative administration and critical care phase exposure. Methodological rigor was confirmed through concordant findings from alternative propensity score implementations and multivariable-adjusted time-to-event analyses.

### Previous clinical evidence

Autonomic effects are crucial in the pathogenesis of both nonsurgical and cardiac surgical AF (Gaudino et al., 2023). Two studies have reported a significant link between dopamine administration and an increased risk of AF following cardiac surgery (Salaria et al., 2005; Argalious et al., 2005). Additionally, one study has documented a similar association between norepinephrine use and elevated AF risk in this population; A randomized controlled trial by Ludhmila et al. (n = 330) demonstrated elevated atrial fibrillation risk following cardiac surgery with norepinephrine administration. This evidence supports the pathophysiological interplay between exogenous adrenergic agents and endogenous catecholamine excess in postoperative arrhythmogenesis. Numerous studies indicate that triggering the sympathetic nervous system during or after surgery with pain-related stimuli (Meijer et al., 2020), surgical injuries, anesthesia type, fluid management, discomfort (Priebe, 2016), and preexisting comorbidities like hypertension (Huggett et al., 2004), heart attack, are associated with cardiovascular incidents (Hering et al., 2015). Not ignorably, patients requiring phenylephrine may inherently have hemodynamic instability, which itself is a risk factor for AF. Currently, the link between sympathetic stimulation and atrial fibrillation (AF) post-surgery remains ambiguous. It was hypothesized that administering phenylephrine during or after surgery could elevate the likelihood of atrial fibrillation, leading us to undertake these findings to explore the connections between phenylephrine and the emergence of this type of AF.

It is critical to emphasize that the observed association between phenylephrine and atrial fibrillation (AF) does not imply that phenylephrine should be universally avoided in postoperative settings. Rather, these findings suggest that clinicians should exercise heightened vigilance when administering phenylephrine to specific patient subgroups, particularly those with pre-existing cardiac risk factors (e.g., hypertension, left atrial enlargement, or diastolic dysfunction) or in clinical scenarios where the risk of AF is already elevated (e.g., cardiac surgery or prolonged hemodynamic instability). This nuanced approach allows for the judicious use of phenylephrine while minimizing potential arrhythmogenic risks.

### Potential possible mechanisms

Phenylephrine, as a selective α1-adrenergic agonist, exerts its primary effects through systemic vasoconstriction, which increases vascular resistance and afterload (Meng et al., 2024; Thiele et al., 2011). This rise in afterload directly impacts left ventricular function, as the heart must generate greater pressure to eject blood into the systemic circulation. Consequently, left ventricular end-diastolic pressure (LVEDP) increases, leading to elevated left atrial pressures due to the backward transmission of pressure through the pulmonary circulation. This hemodynamic cascade results in atrial stretch, a well-documented trigger for structural and electrical remodeling in atrial tissue. Atrial stretch activates mechanosensitive ion channels and alters the electrophysiological properties of atrial cardiomyocytes (Chen et al., 2021). Specifically, it can lead to: Shortening of the atrial effective refractory period (ERP), which increases the susceptibility to re-entrant circuits, a key mechanism underlying AF; Stretch-induced calcium handling abnormalities can promote early afterdepolarizations (EADs) and delayed afterdepolarizations (DADs), which are potential triggers for AF initiation (Kalman et al., 1995; Andrade et al., 2014); Chronic or repetitive atrial stretch can activate fibroblasts, leading to extracellular matrix deposition and atrial fibrosis, which further disrupts electrical conduction and promotes AF persistence. These mechanisms are particularly relevant in the perioperative setting, where patients often experience fluid shifts, sympathetic activation, and inflammatory responses that exacerbate atrial stretch and electrical instability. The pathophysiological triad of elevated ventricular afterload, atrial wall tension, and adrenergic hyperactivation establishes an arrhythmogenic substrate, mechanistically linking phenylephrine exposure to AF pathogenesis through β-receptor-mediated electrophysiological destabilization.

### Limitations

This retrospective analysis presents five limitations warranting attention: First, the nonrandomized cohort architecture introduces residual confounding, necessitating future RCTs to isolate phenylephrine-specific arrhythmogenic effects from therapeutic confounding. Second, Undifferentiated atrial fibrillation classification (paroxysmal/persistent subtypes) and absence of rhythm control metrics restrict pathophysiological interpretation, mandating prospective registries with granular electrocardiographic phenotyping. Third, While β-adrenergic hyperactivation emerges as a plausible pathway, the precise ionic mechanisms linking catecholamine surges to atrial remodeling remain uncharacterized—a knowledge gap addressable through optogenetic murine models and human induced pluripotent stem cell-derived cardiomyocyte assays. Fourth, current sample sufficiency precluded comprehensive comorbidity stratification (e.g., heart failure vs. valvulopathy subgroups), highlighting the imperative for international large-cohorts. Fifth, The observational paradigm cannot discount reverse causation scenarios where undiagnosed arrhythmic substrates prompted vasopressor requirement.

## Conclusion

This cohort analysis establishes phenylephrine as an independent risked predictor of new AF, thereby informing evidence-based vasopressor selection through individualized risk-benefit calculus during hemodynamic management. Such pharmacovigilance insights empower clinicians to optimize catecholamine therapy while mitigating iatrogenic arrhythmogenic potential in critical care contexts.

## Data Availability

The original contributions presented in the study are included in the article/Supplementary Material, further inquiries can be directed to the corresponding authors.

## Abbreviations

AF, atrial fibrillation; PSM, Propensity score matching; CKD, chronic kidney disease; ARB, angiotensin converting enzyme inhibitors; ACEI, angiotensin II receptor blocker; CCB, calcium channel blockers; ORs, odds ratios; CIs, confidence intervals; EADs, afterdepolarizations; Med-Surg ICU, Medical-Surgical ICU; SICU, Surgical ICU; CSICU, Cardiovascular Surgery ICU; CCU-CTICU, Cardiology care unit-Cardiothoracic ICU; CTICU, Cardiothoracic ICU; MICU, Medical ICU; SAPS II, Simplified Acute Physiology; APACHE IV, Acute Physiology and Chronic Health Evaluation IV.
