# Supplementary material for: Phenylephrine and the risk of atrial fibrillation in critically ill patients: a multi-centre study from eICU database
Source: Front Pharmacol. 2025 Mar 26;16:1478961. doi: 10.3389/fphar.2025.1478961 (PMC11979180; doi:10.3389/fphar.2025.1478961)
Supplement: Supplementary file 1 [file DataSheet1.docx]

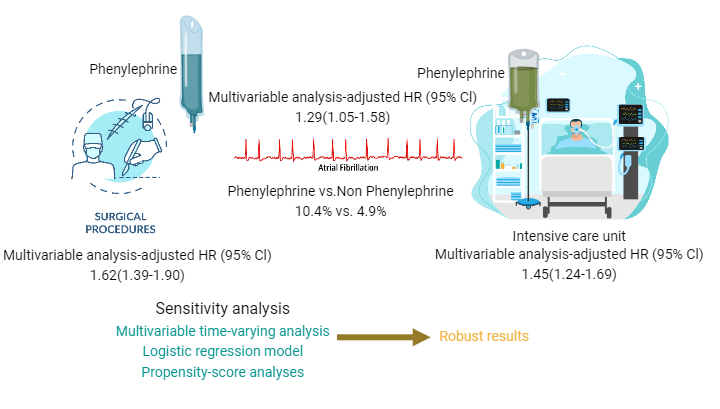


Abstract graph


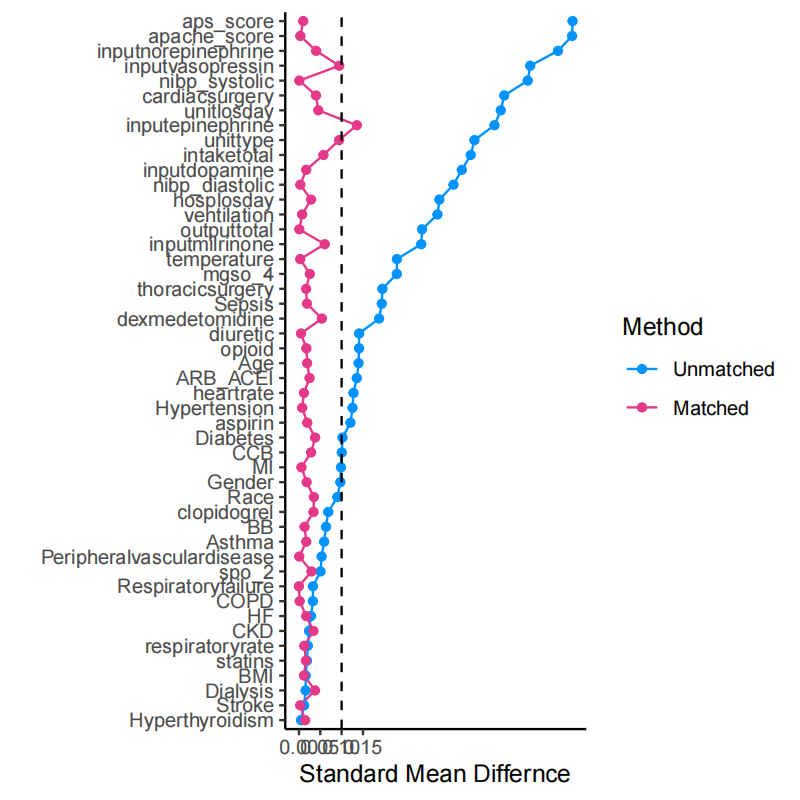


**SFig 1. Absolute Standardized Mean Differences in Individual Covariates Before and After Propensity Score Matching in the entire Cohort**


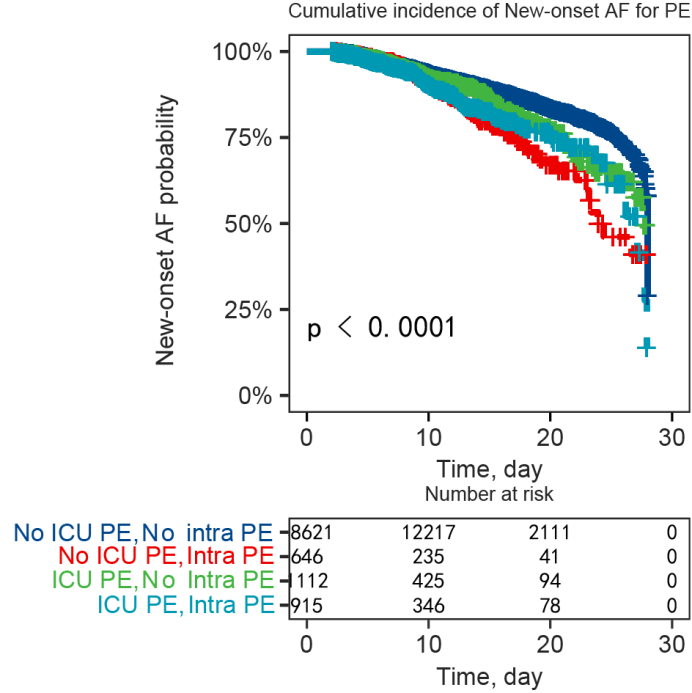


**SFig 2. Cumulative risk of new-onset AF for intraoperative phenylephrine use or/and phenylephrine treatment in ICU**

Abbreviations: PE, phenylephrine. Intra PE was defined as intraoperative phenylephrine use; ICU PE was defined as phenylephrine treatment in ICU.

| Exposure | AF, events | Incidence of atrial fibrillation |
| --- | --- | --- |
| Vasopressin | 139/1389 | 10% |
| Epinephrine | 64/859 | 7.4% |
| Norepinephrine | 494/6776 | 7.2% |
| Phenylephrine | 282/2673 | 10.5% |
| Dopamine | 93/1414 | 6.5% |
| Milrinone | 50/428 | 10.6% |

**STable1. Antihypertensive drugs and the incident of new-onset atrial fibrillation**

**STable2. Comparative associations between Phenylephrine and Norepinephrine Use with New-onset Atrial fibrillation in the Univariate Cox Analysis after Propensity-Score matching**

| New-onset AF | | All | HR (univariable) |
| --- | --- | --- | --- |
| Norepinephrine | No | 1345 (50.0%) |  |
| Phenylephrine | Yes | 1345 (50.0%) | 2.26 (1.69-3.03, p<.001) |
|  | | | |
